# Supplementary material for: The interactome of intact mitochondria by cross-linking mass spectrometry provides evidence for coexisting respiratory supercomplexes
Source: Mol Cell Proteomics. 2017 Dec 8;17(2):216–32. doi: 10.1074/mcp.RA117.000470 (PMC5795388; doi:10.1074/mcp.RA117.000470)
Supplement: Supplemental Data [file supp_RA117.000470_133922_0_supp_23978_fzffwf.zip › spectra_annotation/mito_DR_spectra_annotation/470-1-10-1-12-1.pdf]

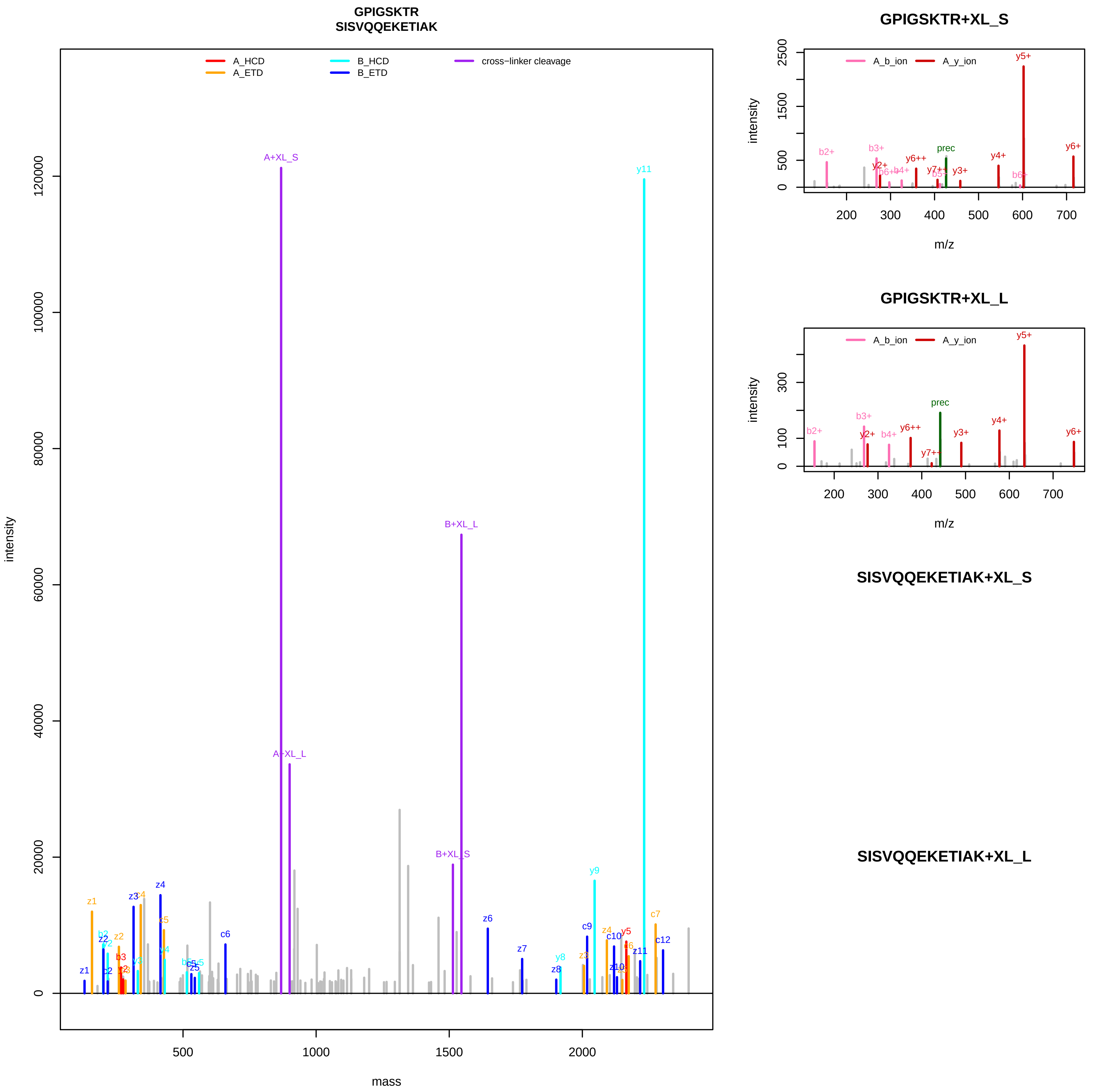
GPIGSKTR
SISVQQEKETIAK
A_HCD
A_ETD
B_HCD
B_ETD
cross−linker cleavage
A+XL_S
A+XL_L
B+XL_L
B+XL_S
y11
y9
y8
y5
c7
c9
c10
c12
c6
z1
z2
z3
z4
z6
z7
z8
z11
intensity
mass
0
20000
40000
60000
80000
100000
120000
500
1000
1500
2000
GPIGSKTR+XL_S
A_b_ion
A_y_ion
b2+
b3+
y2+
y6++
prec
y4+
y5+
y6+
y3+
intensity
m/z
GPIGSKTR+XL_L
A_b_ion
A_y_ion
b2+
b3+
y2+
b4+
y6++
y7++
prec
y3+
y4+
y5+
y6+
intensity
m/z
SISVQQEKETIAK+XL_S
SISVQQEKETIAK+XL_L
